# Revealing the impact of COVID-19 on mental health through machine learning

**DOI:** 10.1093/jamiaopen/ooag013

**Published:** 2026-01-23

**Authors:** Salah Bouktif, Akib Mohi Ud Din Khanday, Ali Ouni

**Affiliations:** Department of Computer Science and Software Engineering, United Arab Emirates University, Al Ain, Abu Dhabi, 15551, United Arab Emirates; Department of Information Technology, Cluster University of Srinagar, Srinagar, Jammu and Kashmir, 190008, India; École de Technologie Supérieure (ETS Montreal), University of Quebec, Montreal, Quebec H3C 1K3, Canada

**Keywords:** COVID-19, depression, anxiety, machine learning, explainable artificial intelligence

## Abstract

**Objective:**

The COVID-19 pandemic caused a major health crisis worldwide significantly impacting mental well-being. In this study, our objective is to assess the resilience of pre-pandemic depression level prediction models when applied to COVID-19 era data. We leverage advanced Machine Learning (ML) and Explainable Artificial Intelligence (XAI) techniques to identify the key factors impacting the shifts in depression levels during the pandemic. We aim to align the later identification with interventions and preparedness for future pandemics.

**Materials and methods:**

We use, in this study, a data-driven methodology using National Health Interview Survey (NHIS) household survey data, explicitly covering the years 2019-2022. The NHIS data is used to build both the pre-pandemic (2019) and COVID-19 (2020-2022) models discussed in our comparative evaluation. Various ML techniques are supported (1) upstream, using feature selection methods to reduce both irrelevance and the high dimensionality of social-nature data, and (2) downstream, by an XAI-based approach to gain insight into the pandemic-associated phenomena that mostly impacted the mental health of individuals. In our empirical experiments, we use over 100 000 entries across the 4 yearly datasets, where we apply an 80%-20% training/testing split for models building and evaluation.

**Results:**

The outcomes of our empirical study show that classifiers trained solely on pre-COVID-19 data performed poorly when applied to COVID-19 era data. Conversely, models retrained on pandemic-specific data demonstrated high performance. In particular, the Random Forest (RF) classifier achieved the best performance, recording an average accuracy of 98.10% across the COVID-19 era datasets. With respect to the depression key factors’ identification, XAI techniques provided actionable insights, revealing that features such as Delayed Medical Care, Family Poverty, Participation in Social Activities, and Marital Status were the most influential factors contributing to depression challenges during the pandemic.

**Discussion and conclusion:**

The significant decline in the performance of pre-pandemic models on COVID-19 data reveals the profound impact of the pandemic on mental health, highlighting the need for new predictive models tailored to crisis circumstances. The built RF model, uses appropriate pandemic data, performed accurately during the COVID-19 era with an accuracy of 98.1%. XAI techniques confirmed that factors such as delayed medical care, family poverty, job loss, and reduced social involvement were critical drivers that impacted the decline in mental health during the pandemic.

## Introduction

Mental health is a cornerstone of human well-being that has been shaped by biological, psychological, and social factors. COVID-19 had a significant impact on mental health worldwide.^1^ During the pandemic, the combination of physical health risks, social isolation, economic stress, and uncertainty has led to increased stress levels, anxiety, and depression.^2^ The pandemic created a range of stressors, including concerns about personal health and safety, fear of contracting the virus or transmitting it to loved ones, concerns about job security, financial difficulties, and disruption of daily routines.^1^ These stressors affected mental health, particularly the level of depression among individuals.^3^^,^^4^ The necessary measures implemented to control the spread of the virus, such as lockdowns, social distancing, and travel restrictions, resulted in increased social isolation and reduced social connections.^5–7^ These pandemic circumstances and others developed a feeling of loneliness, depression, and emotional distress.^2^^,^^3^

Understanding the impact of the COVID-19 related stressors on mental health, particularly depression, is crucial for developing effective interventions to improve the quality and the accessibility of mental health care. This is especially important given that around 280 million individuals worldwide are estimated to suffer from depression.^8^^,^^9^ By predicting the level of depression early and diagnosing the causes, we can potentially prevent subsequent behaviors such as suicidal ideation.^10^

The primary challenge addressed in this research is modeling mental health, specifically the level of depression, using ML techniques. A key question is whether models trained on pre-COVID-19 data can effectively predict depression levels during the COVID-19 pandemic, or if new models should be trained using data collected during the pandemic. This research aims to identify the main features impacting mental health before and during the COVID-19 period and to understand the differences in these features across the 2 timeframes. While proposing a solution to the stated problem, we direct our work towards answering 2 research questions:

- *RQ1:* How do depression models trained on datasets from before COVID-19 perform on the datasets collected during the COVID-19 era?
- *RQ2:* To what extent does COVID-19 impact mental health?

This study proposes the use of machine learning (ML) techniques, supported by feature selection and interpreted by Explainable AI (XAI) methods, to gain insights into the most impactful features affecting depression levels during the 2 timeframes. By investigating the effectiveness of ML models trained on pre-COVID-19 data when applied during the COVID-19 period, we aim to determine whether these models can accurately predict and analyze trends related to mental health during the pandemic and beyond. XAI techniques will be used to identify and understand the key factors contributing to changes in mental health due to the pandemic, providing valuable insights into the impact of COVID-19 on mental health.

This research makes a significant contribution to advancing the understanding and management of mental health, with a particular focus on depression in the context of the COVID-19 pandemic. One key aspect of the study is the evaluation of depression across different timeframes. By assessing the effectiveness of ML models trained on pre-COVID-19 data in predicting depression levels during the pandemic, the research aims to determine whether existing predictive models are robust enough to handle unprecedented situations or if there is a need to develop new models tailored to pandemic-specific data. This evaluation is crucial for understanding the adaptability of current methodologies in the face of global crises.

Another important contribution lies in the identification of key features that impact mental health. Utilizing Explainable AI (XAI) techniques, the study systematically uncovers and interprets the primary factors influencing depression both before and during the COVID-19 period. This approach provides valuable insights into how various stressors and contextual factors affect mental health across these 2 distinct timeframes, thereby enhancing our understanding of the dynamic nature of mental health determinants in response to societal disruptions.

Furthermore, the research aims to improve mental health interventions by harnessing the combined power of ML and XAI. By facilitating earlier detection and more accurate diagnosis of depression, the study supports the development of more effective and accessible mental health care strategies. Ultimately, these advancements have the potential to reduce the prevalence of depression, improve patient outcomes, and contribute to the prevention of suicide, thereby addressing a critical public health concern exacerbated by the pandemic.

In this work, we used National Health Interview Survey (NHIS)^11^ which included questions related to depression, anxiety, and other serious issues. Based on the survey data, ML algorithms were developed to create early warning systems for mental health issues. While prior studies have explored the potential of ML algorithms to monitor online activity—such as changes in behavior, sentiment, or language usage—for early detection of mental health decline,^12^^,^^13^ the majority of existing work remains concentrated on social media data. In contrast, this study advances the field by moving beyond sentiment analysis and online behavior, instead extracting novel features from structured household survey data collected during a major global event—the COVID-19 pandemic. By investigating the impact of diverse sociodemographic and contextual factors on mental illness, our approach highlights the broader applicability of ML in mental health diagnostics and supports the development of more inclusive, data-driven early warning systems. Moreover, XAI techniques were used to identify the factors that impact mental health. The findings revealed that the models trained on pre-COVID-19 data do not perform well on COVID-19 and post-COVID-19 Data. When using XAI techniques, various factors in the COVID-19 era were identified to be impactful for mental Health (ie, depression level). It was found that Delayed Medical Care, Family Poverty, Age, Participation in Social Activities, and Marital Status are the important factors that lead to mental health problems. Through this approach timely interventions/support can be provided, which in turn will significantly reduce mental health problems. It may not only improve the lives of those directly affected but also can have a positive effect on society, which may lead to reduced healthcare costs, increased productivity, and the promotion of a more compassionate and inclusive social environment.^12^

The overall structure of the paper consists of 5 sections, In Related Work section, the related work about mental health, COVID-19, and ML is discussed. Methodology section discusses the methodology used to analyze data related to mental health. Discussion section discusses the results generated after implementing the proposed approach and Conclusion section concludes our work.

## Related work

In this section, we discuss the landscape of related work in mental health diagnosis, exploring key studies, methodologies, and trends that have shaped this dynamic and critical field. We examined how these approaches have evolved, the challenges they address, and the potential they hold for more effective and accessible mental health diagnostics.

According to the Diagnostic and Statistical Manual of Mental Disorders (DSM) published by the American Psychiatric Association (APA), depressive disorders encompass a range of diagnostic categories—such as Major Depressive Disorder, Persistent Depressive Disorder, and others—defined by specific symptom patterns. In addition to these categorical distinctions, depression is also clinically assessed based on severity levels, typically classified as mild, moderate, or severe. This severity-based classification forms the central focus of our study, as we aim to model and predict depression intensity using ML techniques applied to survey data.^13^ The DSM provides broad criteria for classifying mental disorders based on observed symptoms. When an individual exhibits at least one of the following 2 symptoms: Persistently depressed mood most of the day or loss of interest or pleasure. The process of diagnosing depression is frequently hindered by the possibility of misidentification, its time-intensive nature, and the reality that not all individuals with depression openly display typical depressive symptoms, such as feelings of helplessness or hopelessness.^14^^,^^15^ The interplay of biological factors, family and environmental stressors, and individual susceptibilities significantly influences the development of Major Depressive Disorder (MDD).^16^^,^^17^ It is widely recognized that the way depression is represented can be influenced by various factors.^18^^,^^19^ Traditionally, psychiatric diagnosis has relied heavily on what patients report about their symptoms and how they respond during clinical interviews—an approach formalized in frameworks like the DSM-5.^20^ This emphasis on subjective reporting stems from the absence of universally accepted biomarkers and the complex, individualized nature of mental health conditions. Observable behavioral cues, while present, have not been considered primary indicators in clinical practice. However, recent advances in wearable technologies and ML have opened new possibilities. These tools enable the continuous capture of real-world behavioral data, leading to the development of smartphone-based diagnostic systems for conditions like depression. Building on earlier research, these systems show that objective physiological, biological, and behavioral indicators can significantly improve diagnostic accuracy and may help reduce the broader socioeconomic burden of mental illness.^20^  Table 1 summarizes some recent works related to the mental health (ie, level of depression).

**Table 1.** Some recent works related to the mental health (depression).

Dataset used	Contribution	Results	Research gap	References
Twitter(2.5 M)	Machine learning classifiers analyze Twitter data to detect depression through linguistic histories	Naive Bayes achieves impressive ROC AUC (0.94) and precision (0.82) with 86% accuracy	Only Twitter is used for collection of depression related posts.	^21^
LiveJournal, Twitter, and Facebook	Forecasting Depression Levels from Social Media Posts via ML Classifier.	SVM outperformed, achieving 57% accuracy, 67% precision, and 56% recall.	Only the Sentimental feature is used for classification	^22^
Facebook Comments	The authors employed ML techniques on publicly available Facebook data to detect depression.	Decision Tree outperforms other ML algorithms.	More data heterogeneity may improve the performance	^23^
Facebook data(150 045)	Detected Facebook users’ moods using the fine-tuned KNN (k-nearest neighbors) classification algorithm.	The best-performing fine-tuned model among the several KNN classifiers was Coarse KNN, which had a high F-score	Other social media platforms may be used for collecting the relevant dataset	^24^
Twitter	Uses ML-based approaches to find whether a Twitter user is depressed or not based on his/her social network behavior and tweets.	SVM-linear classifier demonstrated the best performance among other ML-Classifiers	More feature engineering may increase the performance of the models	^25^
53 Volunteers	Detected depression using RBFN with text and speech inputs, utilizing the Partial Least Square algorithm for vocal expression-based detection.	An Accuracy of 71.4% is achieved.	More Data can be supplied to improve efficiency	^26^
671 participants (Interviews)	Detected depression by combining deep multimodal neural networks with a purpose-built automated evaluation.	The results showed the Sensitivity of 68.59%, Specificity of 67.46%, and F-Score of 67.66%	More survey diversity may contribute to better results.	^27^
Chatterbot Corpus	Proposed an Expert System for Stress Management to provide the best recommendation and solution for youth, especially IT professionals	Chatbot Recommender system based on Naive Bayes technique.	Other datasets may be used for investigating more with the use of state of art NLP techniques	^28^

While recent advances in wearable technologies and ML have opened new avenues for objective mental health assessment, the broader application of data mining in predicting and preventing mental illness remains limited. Our literature survey revealed that this area is still in its early stages and demands substantial effort to overcome key challenges. Chief among these is data scarcity—driven by the sensitive nature of mental health information and the ethical constraints surrounding its collection and use. These limitations have slowed progress in developing scalable, data-driven diagnostic tools. The following research gaps highlight the need for more robust methodologies and richer datasets to fully realize the potential of ML in mental health care. The following research gaps underscore the need for more inclusive methodologies and richer datasets to fully realize the potential of ML in mental health care:

1. Overreliance on social media data limits the generalizability of findings.
2. Feature selection in current models often overlooks high-impact variables beyond sentiment and language.
3. Structured surveys provide a more reliable foundation for identifying mental health trends, disparities, and intervention opportunities.

## Methodology

**Figure 1.**
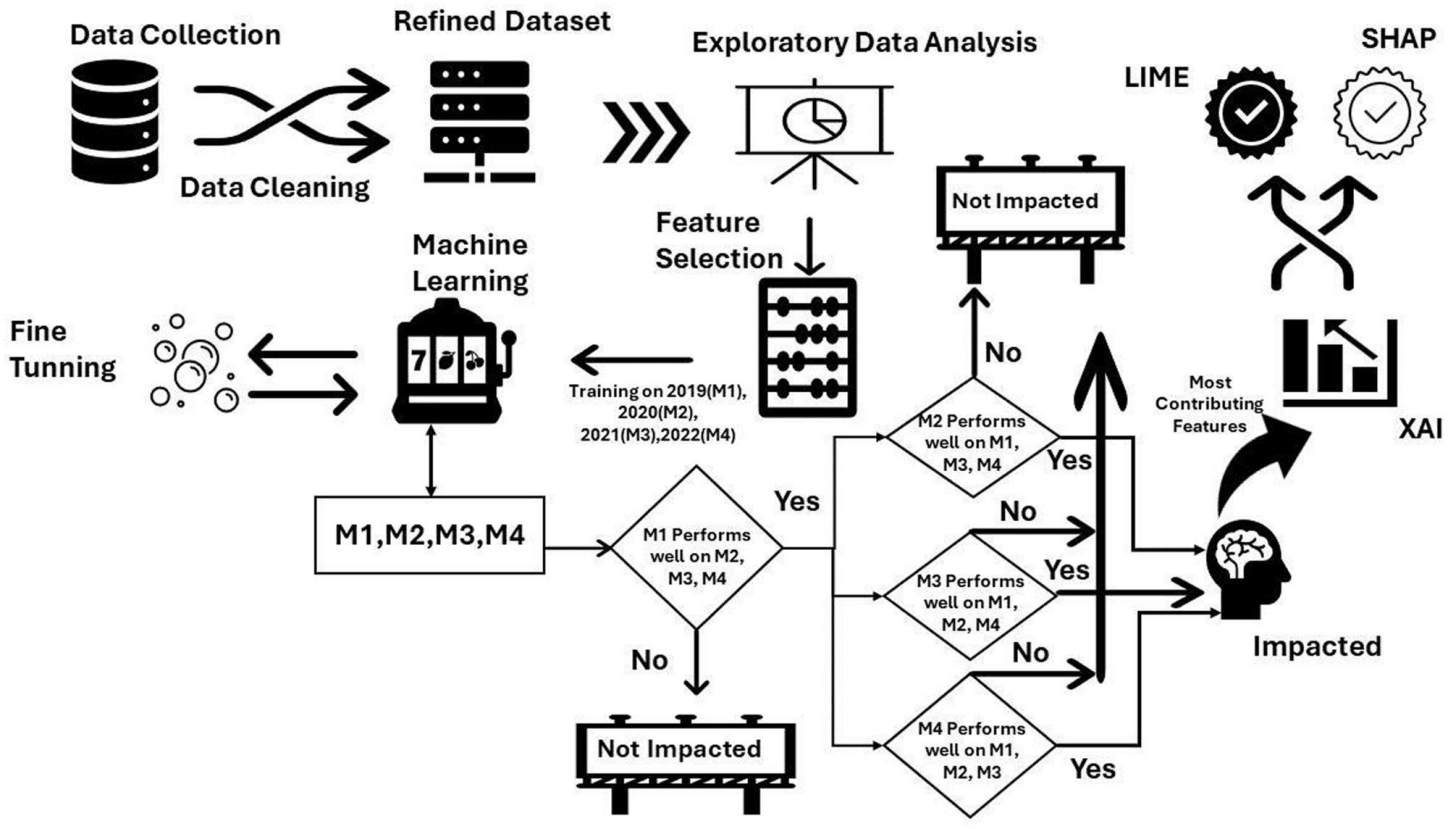
Overall framework for the mental health analysis.

We adopt a data-based approach to analyze mental health using ML algorithms. Our approach consists of 5 stages as shown in Figure 1. Data were collected from the household surveys^11^ and labeled by the level of depression severity that is classified into 3 distinct classes: Severe, Moderate, and Low. We refined data in a preprocessing stage and used feature engineering to reduce features using dimensionality reduction approach.^29^ Thereafter, we classify data using various ML algorithms. Finally, we used explainable AI techniques based on Local Interpretable Model-agnostic Explanations (LIME) and SHapley Additive exPlanations (SHAP) to identify the most contributing factors.

### Data collection

**Figure 2.**
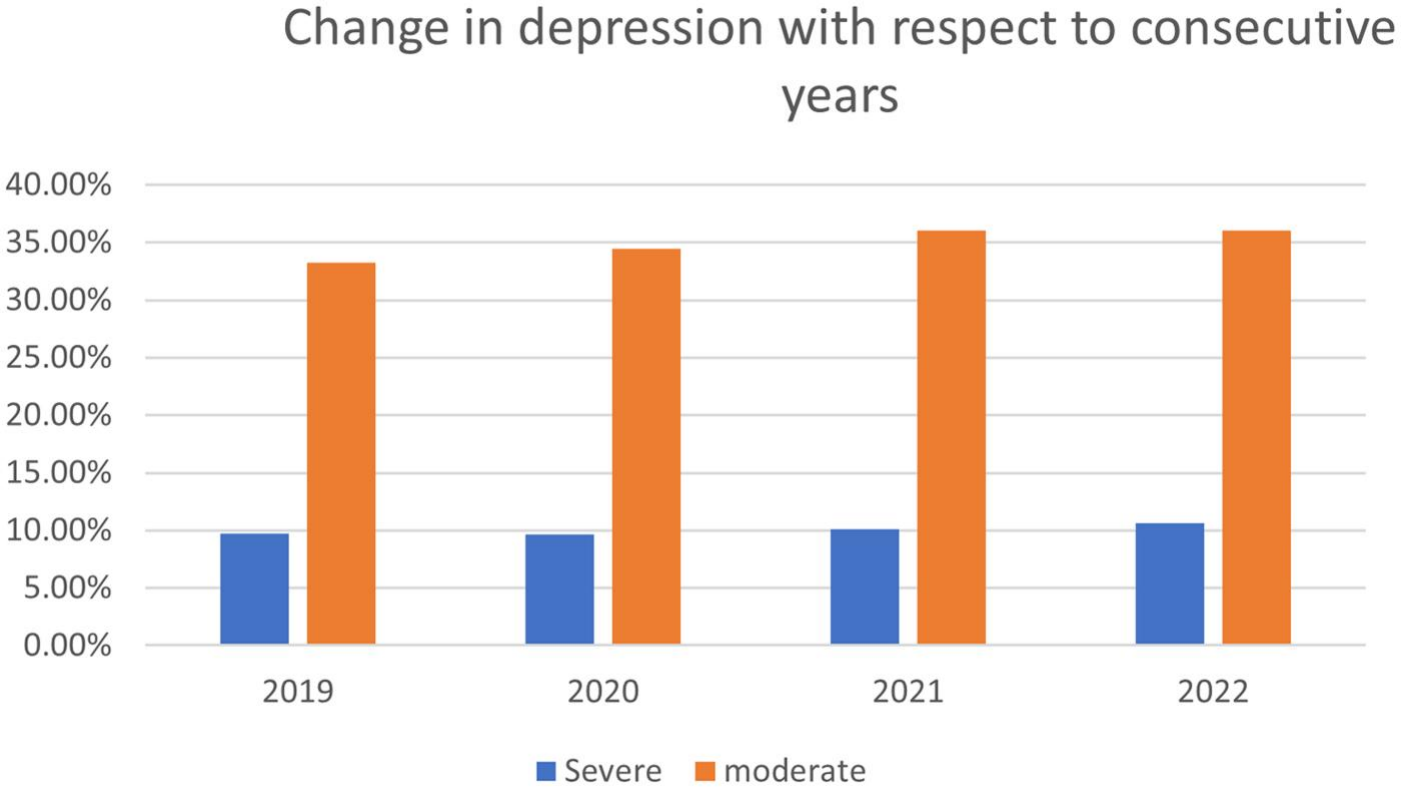
Change in depression percentage from 2019 to 2022.

We collected data from NHIS.^11^ We conducted separate analyses, first with data from the 2019 NHIS to train the baseline model, then calibrating the model and testing on the 2020, 2021, and 2022 NHIS datasets. The description of the datasets used is shown in Table 2. All 4 datasets under investigation offer a comprehensive assessment of the health status of the adult population in the United States. The corresponding changes in the level of depression in terms of severity across these 4 years are shown in Figure 2.

**Table 2.** Description of the datasets used for analysis.

Dataset	Attributes	Entries	Attributes related to mental health	Low level	Moderate level	Severe level
2019	533	31 997	14	9.74%	33.27%	56.99%
2020	616	31 568	13	9.67%	34.47%	55.86%
2021	621	29 482	20	10.09%	36.05%	53.86%
2022	636	27 651	21	10.60%	36.05%	53.36%

### Data preprocessing

In this phase, the “SimpleImputer” method was leveraged for handling missing and null values. Also, duplicate data were removed, and data normalization was performed, which prevents certain features from dominating the analysis due to their inherent scale.

### Feature engineering

Feature selection was performed to identify the most relevant variables from the dataset including mental health indicators, socioeconomic factors, and COVID-19-related aspects. Since the datasets used in this study are diverse and have different numbers of features. Table 2 shows the number of features in each dataset with the number of questions related to mental health. Principal Component Analysis (PCA) was used, which transforms high-dimensional data into a lower-dimensional form while preserving the essential information in the original dataset.

In this study, 200 principal components were selected by using the PCA library in Python. The decision to select 200 principal components in the feature selection process was based on achieving a balance between dimensionality reduction and information retention. These 200 components were chosen because they capture a sufficient proportion of the total variance in the dataset. This choice allowed for a significant reduction in the feature space which improves computational efficiency while minimizing information loss. These principal components were selected from each dataset. After performing PCA, we had 4 datasets each with 200 attributes(principal components) and a target label of depression (Severe, Moderate, and Low).

### Classification

Our classification step aims at building classifiers to categorize the level of depression severity into Low, Moderate, and Severe. Therefore, we fine-tuned 3 classifiers: Support Vector Machine (SVM), Random Forest (RF), and Artificial Neural Networks (ANN). SVM is effective in high-dimensional spaces and non-linear decision boundaries, making it useful for complex classification tasks. RF provides robustness by handling both numerical and categorical features well, offering feature importance insights. Neural Networks excel at capturing intricate patterns in large datasets which make them ideal for more sophisticated modeling. By fine-tuning and comparing these models we aimed to identify the appropriate classification technique for accurate depression level predictions. As evaluation metrics, we used precision, recall, *F*-score and accuracy. To achieve the best performance for intra-year classification, where models are trained and tested on the same year’s data, we leveraged a sophisticated feature engineering and fed our classifiers with a selected set of features. By training and testing depression classifiers on data of the same year, we identified that RF model demonstrated a powerful and consistent synergy with high accuracy for the intra-year classification. The achieved performances of the classifiers are reported and discussed in the result section.

The overall approach is shown in Algorithm 1.

Algorithm 1  Depression detection using Machine Learning

**Require:** Survey Data $\left(S_{\mathrm{input}}\right)$, Classifier_Name, Hyperparameters

**Ensure:** Severe Depression $\left(D_{s}\right)$, Moderate Depression$\left(D_{m}\right)$ and Low Depression $\left(D_{l}\right)$

1: **START** 

2: **for** *i from* 1 *to n* **do** 

3:  *C[i]* = $S_{\mathrm{input}}[i]$ + *Label*

4: **end for** 

5: *Dep* = *C[i]*

6: *Dep* = NR(Dep)//Removing Null Values

7: *Dep* = HM(Dep)//handling Missing Values

8: *Processed*.*csv* = *Dep*//Saving Preprocessed file as CSV

9: *Feature[i]* = Feature(Dep)//Feature Engineering and Selection

10: Optimal_Features = *Feature[i]*//dimensionality reduction

11: **for** d from 1 to 4 **do** 

12:   *CLASSIFIER* (Classifier_Name,Hyperparameters,  Cross_Valid=10,Optimal_Features,dataset[d])

13: **end for** 

14: whether COVID-19 impacted or Not

15: **END** 

## Results and discussion

The experiments were performed on Google Collab. The yearly classifiers were trained and tested based on an 80-20 ratio where 80% of data was used for training a model and 20% of data was used for testing. We remind that each classifier was trained and tested on data of the same year using 10-fold cross validation. The fine-tuned RF, SVM, and Neural Network classifiers all demonstrated high performance intra-year evaluations when their training and testing data were sourced from the same year. In particular, the accuracies of the “yearly” RF classifiers were 97.96% for the 2019 classifier, 97.5% for the 2020 classifier, 98.03% for the 2021 classifier, and 97.79% for the 2022 classifier. The 2019 RF classifier was particularly robust, also achieving 98% precision, recall, and *F*-score. In contrast, the SVM, using the same derived features, lagged significantly, with its accuracy peaking at 82.76% for the 2019 SVM and falling as low as 79.8% for the 2022 SVM. The details on the performance of all the built classifiers including Neural Networks are shown in Table S1 in the supplementary material of this paper.

The core objective of this work is to evaluate the effectiveness of pre-pandemic depression models (built on data collected before COVID-19 era) on COVID-19 data and identify the crucial factors impacting mental health during the pandemic. For this goal, we have formulated 2 hypotheses stating the followings:

1.
$H_{1}$ : The performance of Pre-COVID-19 models is low when used on COVID-19 datasets.
2.
$H_{2}$ : COVID-19 significantly impacts mental health.

### Hypothesis testing

We remind that our empirical experiments use over 100 000 entries across the 4 yearly datasets as detailed in Table 2. Each depression model is trained on the entire data of a specific year, and evaluated on the entire data a different year. To provide a clearer description of the Hypothesis H1 testing, the settings of model training and evaluation are summarized in Table 3. Hypothesis H1 testing is performed on the 3 types of classifiers (SVM, RF, and ANN). For instance, the first row in Table 3 shows the setting of the training and evaluation of the 2019 classifiers (pre-COVID-19 classifiers). They are trained on the whole Pre-COVID-19 data collected in 2019 (31 997 entries) and then evaluated on data collected in 2020 (31 568 entries, during COVID-19 data) to test their performance during COVID-19 era, which indeed showed lower performance as can be seen in Table 4. The subsequent rows in Table 3 detail the various cross-year evaluations settings conducted for models specifically trained and tested on data within the COVID-19 era (2020-2022), demonstrating their higher performance (eg, RF built on during-COVID-19 data, achieving 98.10% accuracy on average in the COVID-19 era). Specific purposes of 7 experiments are outlined in Table 3 and the obtained results are presented in Table 4.

**Table 3.** Experimental setup for hypothesis testing.

Hypothesis/model evaluation focus	Model era (model type)	**Training data year** (Size)	**Evaluation data year(s)** (Size)	Purpose and relation to findings
**H1: low performance of Pre-COVID-19 models on COVID-19 datasets**	Pre-COVID-19 Classifier (SVM, RF, ANN)	2019 (31 997)	2020 (31568)	**Directly tests H1:** Assesses if models trained on pre-pandemic data perform poorly when applied to COVID-19 era data, revealing the pandemic’s significant impact on mental health. (Results reflected in Table 4a)
**Evaluation of COVID-19 era models**	During-COVID-19 Classifier (SVM, RF, ANN)	2020 (31 568)	2021, 2022 (29482), (27651)	Evaluates the performance of models trained and tested within the COVID-19 era, crucial given the observed shifts in depression prevalence and dynamics. (Results reflected in Table 4b and c)
	During-COVID-19 Classifier (SVM, RF, ANN)	2021 (29 482)	2020, 2022 (31568), (27651)	Further evaluates COVID-19 era model performance across different pandemic years to ensure robust understanding of mental health impacts. (Results reflected in Table 4d and 4e)
	During-COVID-19 Classifier (SVM, RF, ANN)	2022 (27 561)	2020, 2021 (31568), (29482)	Completes the evaluation of COVID-19 era model performance, demonstrating the effectiveness of models built with appropriate pandemic data. (Results reflected in Table 4f and g)

**Table 4.** Classification results of all the classifiers.

Classifier	Precision	Recall	*F*-score	Accuracy
SVM	28%	53%	37%	53.35%
RF	88%	88%	88%	87.5%
MLP	74%	75%	74%	74.9%
(a) Trained on 2019 and tested on 2020				
Classifier	Precision	Recall	*F*-score	Accuracy
SVM	97%	96%	96%	96.49%
RF	99%	99%	99%	98.5%
MLP	97%	97%	97%	97.45%
(b) Trained on 2020 and tested on 2021				
Classifier	Precision	Recall	*F*-score	Accuracy
SVM	81%	82%	81%	81.5%
RF	99%	99%	99%	99.6%
MLP	92%	91%	89%	90.7%
(c) Trained on 2020 and tested on 2022				
Classifier	Precision	Recall	*F*-Score	Accuracy
SVM	97%	97%	97%	96.%
RF	98%	98%	98%	98.25%
MLP	98%	98%	98%	97.5%
(d) Trained on 2021 and tested on 2020				
Classifier	Precision	Recall	*F*-score	Accuracy
SVM	97%	97%	97%	96.6%
RF	98%	98%	98%	98.16%
MLP	92%	90%	90%	90.1%
(e) Trained on 2021 and tested on 2022				
Classifier	Precision	Recall	*F*-score	Accuracy
SVM	97%	96%	96%	96.48%
RF	98%	98%	98%	98.2%
MLP	97%	97%	97%	97.2%
(f) Trained on 2022 and tested on 2020				
Classifier	Precision	Recall	*F*-score	Accuracy
SVM	97%	97%	97%	96.64%
RF	98%	98%	98%	98.01%
MLP	97%	97%	97%	97.3%
(g) Trained on 2022 and tested on 2020				

**Figure 3.**
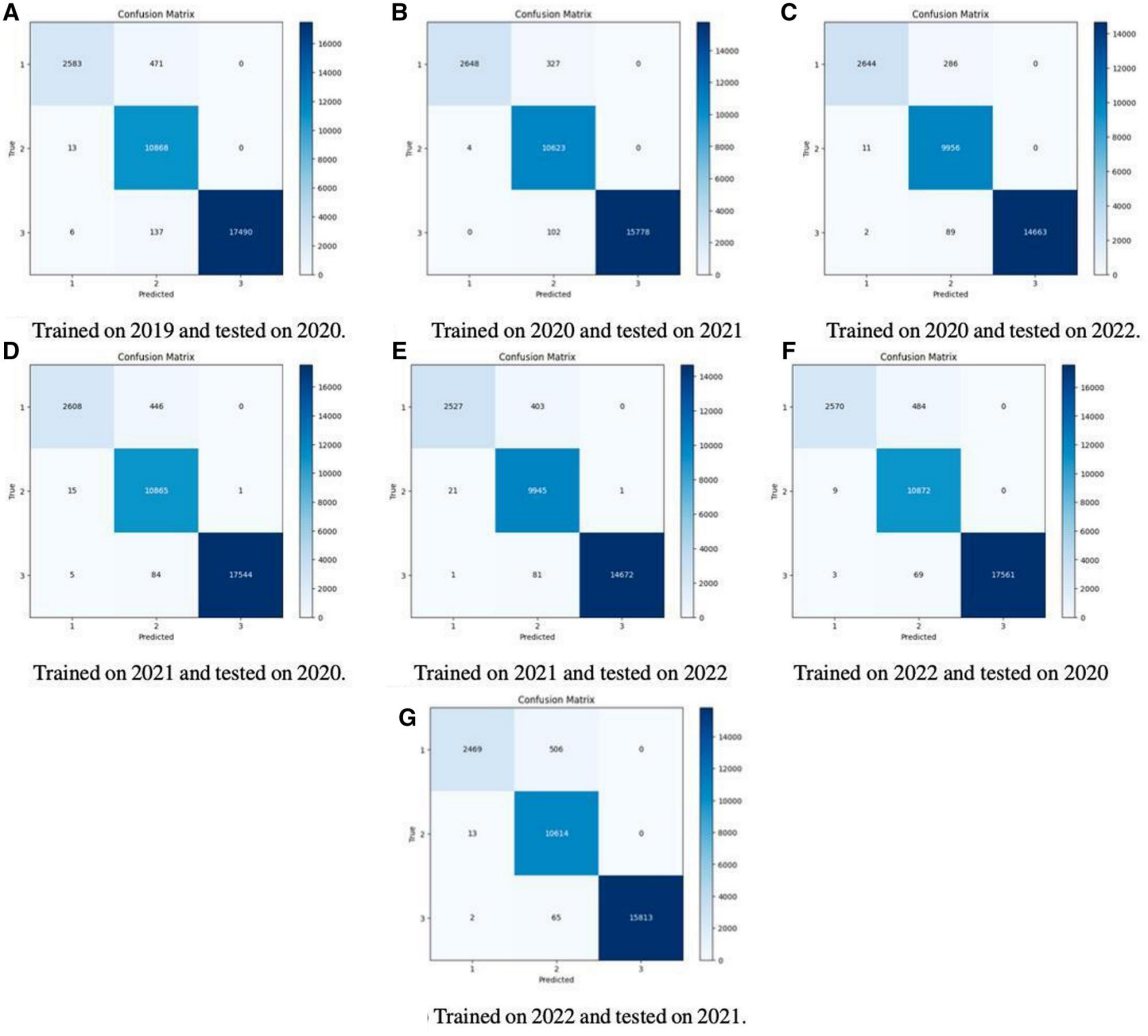
Confusion matrices of the best models.

Indeed, the results revealed that the RF classifier outperformed all other tested models in the context of the COVID-19 era by achieving an average precision of 98.3%, recall of 98.2%, F1-score of 98%, and accuracy of 98.10%. From the experimental results it can be concluded that depression has undergone notable shifts in its prevalence and dynamics in the wake of the COVID-19 pandemic. Pre-COVID-19, depression was characterized by established risk factors such as genetics, trauma, and chronic stress.^30^^,^^31^ In the COVID-19 era, depression has become more complex, influenced by a combination of pandemic-related factors which, as identified by our study’s Explainable AI analysis, include Delayed Medical Care, Family Poverty, Participation in Social Activities, and Marital Status (further detailed in the Discussion section).To check the impact of COVID-19 on mental health, we trained and tested ML classifiers on pre-COVID-19 and COVID-19 data. The best-performing classifier from the pre-COVID models was tested on the COVID-19 datasets respectively and it showed that the models trained on the pre-COVID-19 data do not perform well on COVID-19 datasets as represented by the confusion matrices of the best models in Figure 3. The comparison is shown in Table 4. In addition, the statistical analysis was calculated for the pre-COVID-19 and during-COVID-19 Classifiers. The 2-tailed T-test was performed on the results generated by these classifiers, which showed that null hypothesis stating that the models respectively trained, on pre-COVID-19 data and on during-COVID-19 data, perform similarly, is rejected with confidence of more than 95%, and corresponding statistical results are shown in Table 5.

**Table 5.** Statistical analysis.

Statistics	Pre-COVID	COVID
Mean	87.02	98.213
Variance	1.228444	0.262134
Observations	10	10
Pearson coefficient	0.438871	
*P*-value	5.48 E−11	

### Validation

**Figure 4.**
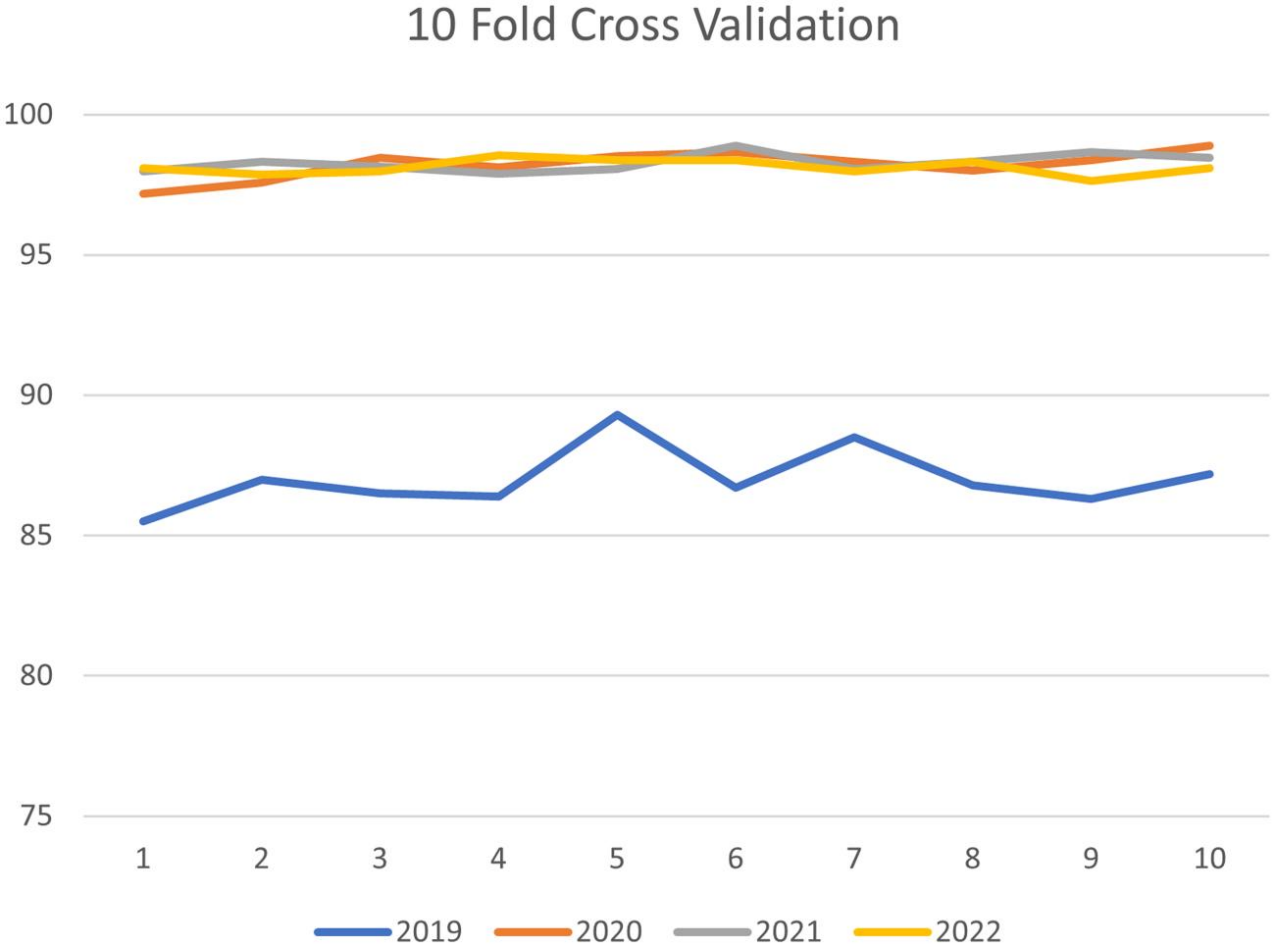
10-fold cross-validation of RF model performance.

To assess the best model’s ability (ie, RF classifier) to generalize from the training data to unseen data and mitigate the risk of overfitting, we conducted a 10-fold cross-validation. The outcomes of this analysis indicated that there was no issue of either underfitting or overfitting during the training and testing of our proposed RF model. In 2019, the average accuracy was 87%, while it significantly increased to 98.21% in 2020, 98.28% in 2021, and remained stable at 98.20% in 2022. Each fold demonstrated a similar trend with accuracy values increasing from 2019 to 2020 and maintaining high performance in the following years. The results of 10-fold cross-validation of the performance of the RF model are shown in Table S2. The graphical representation of these results is shown in Figure 4.

## Discussion

**Figure 5.**
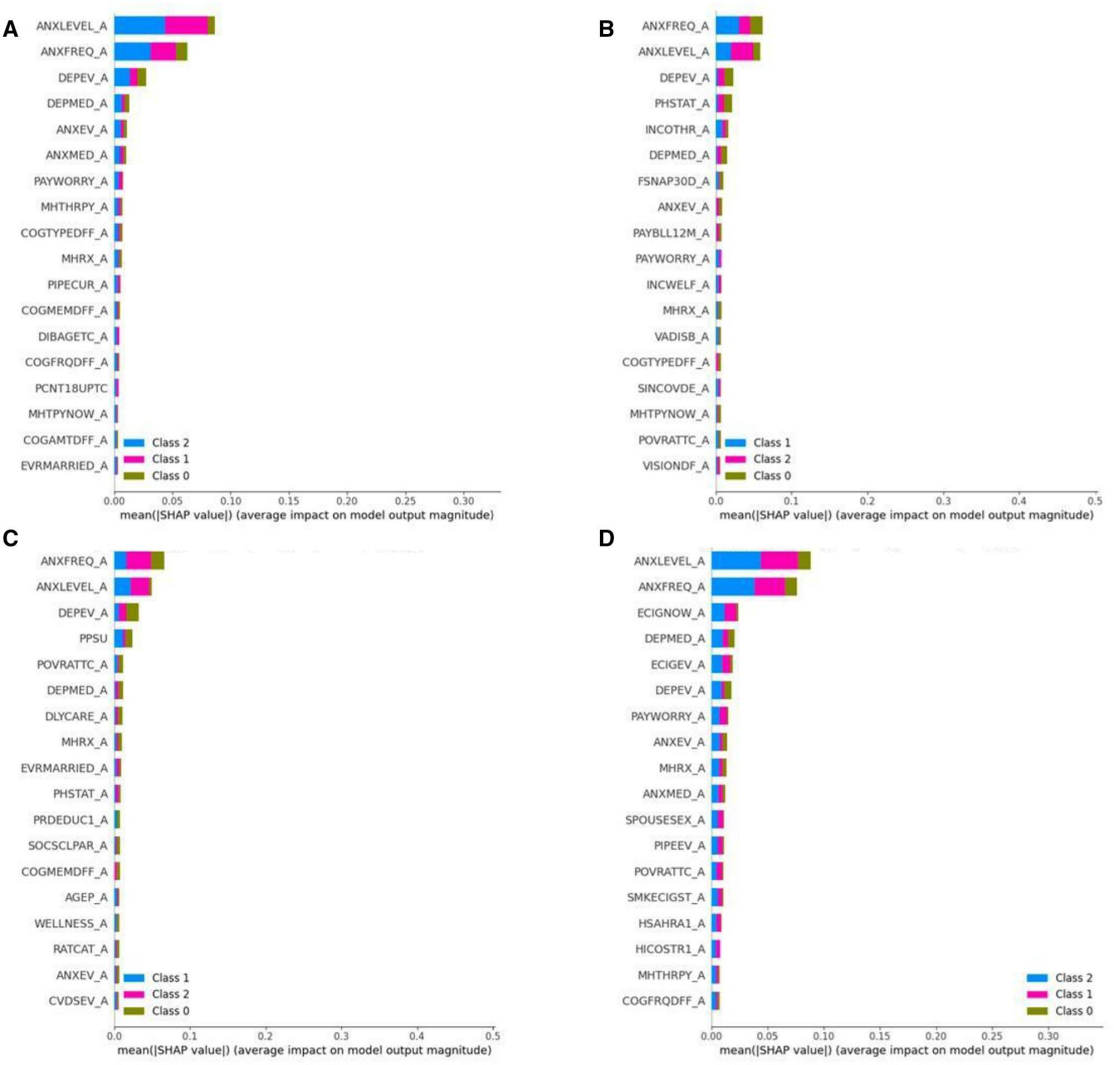
Features generated by the SHAP.

Our findings revealed a significant increase in the prevalence of depression symptoms, attributed to factors such as social isolation, economic stressors, and fear of infection. This analysis shed light on the complex interplay between a public health crisis and mental well-being, emphasizing the need for targeted interventions and support systems to address the long-lasting impact of the pandemic on depression. The different classifiers were trained and tested on the respective datasets. When the classifiers trained on pre-COVID-19 datasets were tested on datasets during COVID-19, it was found that the models did not perform well that much performance. Therefore, it can be concluded that the models trained on 2018, 2019 datasets do not work on 2020, 2021, and 2022 datasets. The results indicate that COVID-19 has affected mental health, due to which the models trained on datasets before COVID-19 do not show better results when testing on datasets collected during COVID-19. The change was identified based on XAI algorithms. XAI enhances human-AI collaboration, facilitates regulatory compliance, and fosters trust in AI technologies.^32^^,^^33^ In this work, we used SHAP and LIME techniques to identify the most contributing factors. Figure 5 shows the most contributing features using the Explainable Boosting Classifier. As depicted in Figure 5, the model trained on pre-COVID-19 has a different set of features that are most impacting the level of depression compared to the models trained on COVID-19 data. The newly identified features are reflecting COVID-19 implications, namely, Delayed Medical Care, Reduced Involvement in Social Activities, Marital Status. These results are explained by the high performance of the depression model (98.1% accuracy), which was achieved when using the new COVID-19-related circumstances impactful factors. These features, which are exclusively impactful during the COVID-19 time and identified among the top features prove that COVID-19-Conditions impact the level of depression, which justifies our hypothesis $H_{2}$.

Table S3 shows the most contributing features with their description. The results generated by the LIME and SHAP give insights into the factors that are responsible for mental health disorders. The results showed that there are various factors that occurred due to the pandemic and triggered mental health issues. Factors that were identified by XAI techniques need to be considered for interventions and preparedness for future pandemics. They include:

1. **Delayed medical care:** Individuals with chronic illnesses or those in need of regular medical attention have faced difficulties in accessing healthcare services and led to stress and anxiety. The fear of visiting healthcare facilities or the unavailability of non-COVID medical services during the pandemic has further strained mental health. Therefore, there is a need for strategies to maintain essential healthcare services during a pandemic and reduce the impact of delayed care on public health, which leads to mental health disorders.
2. **Family poverty:** Poverty leads to social isolation and a lack of access to supportive social networks, negatively affects the mental well-being of families. The inability to afford nutritious food can lead to malnutrition, which is another cause of mental health disorders. Families in poverty face barriers to accessing healthcare, including mental health services, making it challenging to receive treatment for depression.
3. **Age:** Adolescents and young adults have encountered distinct challenges stemming from disruptions in their educational routines, social lives, and an uncertain outlook. The sudden shift to remote learning and reduced social interactions has given rise to feelings of seclusion, anxiety, and depression within this age group. Middle-aged individuals have grappled with the unique demands of tending to both younger and older family members, leading to added stressors. On the other hand, older adults have faced heightened levels of anxiety due to concerns about severe illness or mortality from the virus. Lockdown and restrictions have contributed to social isolation among the elderly, which further affect their mental well-being.
4. **Participation in social activities:** Due to the implementation of social distancing measures, individuals have encountered a reduction in opportunities for in-person social interactions. This includes activities like gatherings with friends and family, participation in community events, and engaging in group activities. The consequences of being unable to take part in these social engagements have resulted in emotions of seclusion, isolation, and a decrease in social support. Many individuals have expressed heightened levels of stress, anxiety, and depression. For certain individuals, the absence of social activities has translated into missing significant life events like weddings, birthdays, and graduations that led to emotional distress.
5. **Marital status:** Marital status has influenced mental health during COVID-19 in complex ways. While marriage can provide emotional and practical support, it also comes with its own set of stressors. Unmarried individuals faced more acute challenges related to isolation and lack of support. Individual circumstances, the quality of relationships, and personal resilience have played a critical role in determining the impact of marital status on mental health during the pandemic.

## Conclusion

COVID-19 has a long-lasting impact on day-to-day life. Mental health is one of the major concerns that has been affected by this deadly virus due to issues like Social distancing, Isolation, Loss of Job, and economic crisis, etc Identifying mental health disorders using household data will facilitate the provision of telehealth services, making mental health support more accessible to those in need. In this work, we analyzed the data from the National Health Survey, which was collected from household interviews. Four different datasets were used, 2019, 2020, 2021, and 2022, respectively. The main motive of this study was to investigate the impact of COVID-19 on mental health using ML. The classifiers were trained and tested on each dataset separately. To verify the impact of COVID-19 the trained classifiers are tested on pre-COVID-19 datasets and COVID-19 Datasets. The results showed that pre-COVID-19 models do not perform well on COVID-19 datasets, which concluded that there is a role of COVID-19 in mental health disorders. Also, the LIME and SHAP were used to extract the most contributing factors that are affecting mental health, and various factors have been added during COVID-19 which triggered mental health into a more severe direction. The results generated by the XAI techniques showed that Job Loss, Delayed Medical Care due to COVID-19, and Reduced Involvement in social activities are affecting the mental health of a person. Countermeasures are needed to tackle the curse of depression, as this deadly health disorder is the main cause of suicide and other harmful activities. In the future, a diverse dataset based on different demographic locations can be used to train and test the models.

## Supplementary Material

ooag013_Supplementary_Data

## Data Availability

The data underlying this article is available via the link provided in data collection section or can be requested to akib.khanday@cusrinagar.edu.in
