# Supplementary material for: Revealing the impact of COVID-19 on mental health through machine learning
Source: JAMIA Open. 2026 Jan 23;9(1):ooag013. doi: 10.1093/jamiaopen/ooag013 (PMC12918303; doi:10.1093/jamiaopen/ooag013)
Supplement: ooag013_Supplementary_Data [file ooag013_supplementary_data.zip › Supplementary.pdf]

Supplementary

October 2025

1 Tables

Table S1: Classification Report of 2020, 2021, and 2022 Datasets

Year	Classifier	Precision	Recall	F-Score	Accuracy
2020	SVM	80%	80%	79%	80.2%
	RF	98%	98%	97%	97.5%
	MLP	32%	56%	41%	56%
2021	SVM	81%	82%	81%	81.5%
	RF	98%	98%	98%	98.03%
	MLP	14%	37%	20%	36.7%
2022	SVM	80%	80%	79%	79.8%
	RF	98%	98%	98%	97.79%
	MLP	40%	36%	20%	36.43%

Table S2: Cross-Validation Accuracy of best model (Random Forest) for Each Fold

Folds	2019	2020	2021	2022
1	85.5	97.18	97.99	98.18
2	87	97.59	98.33	97.86
3	86.5	98.47	98.16	97.97
4	86.4	98.13	97.89	98.65
5	89.3	98.51	98.06	98.57
6	86.7	98.66	98.88	98.37
7	88.5	98.32	98.06	97.97
8	86.8	98.0	98.33	98.53
9	86.3	98.38	98.67	97.74
10	87.2	98.89	98.47	98.18
Average	87	98.21	98.28	98.20

Table S3: Most Contributing Features with their Description

Feature	Description
ANXFREQ_A	How often feel worried, nervous, or anxious
ANXLEVEL_A	Level of feelings when last felt worried/nervous/anxious
DEPEV_A	Ever had depression
PPSU	Pseudo-PSU for public-use file variance estimation
POVRATTC_A	SA family poverty ratio (top-coded)
DEPMED_A	Take medication for depression
DLYCARE_A	Delayed medical care due to COVID-19
MHRX_A	Took medicine for other emotions/concentration/behavior/mental health, past 12m
EVRMARRIED_A	Sample adult has ever been married
PHSTAT_A	General health status
PRDEDUC1_A	Deductible - plan 1
SOCSCLPAR_A	Difficulty participating in social activities
COGMEMDFF_A	Difficulty remembering/concentrating
AGEP_A	Age of SA
WELLNESS_A	Was last visit a wellness visit
RATCAT_A	Ratio of family income to poverty threshold for SA's family
ANXEV_A	Ever had anxiety disorder
CVDSEV_A	Symptoms of COVID-19
